# Associations Between Morning Salivary and Blood Cortisol Concentrations in Individuals With Obstructive Sleep Apnea Syndrome: A Meta-Analysis

**DOI:** 10.3389/fendo.2020.568823

**Published:** 2021-01-19

**Authors:** Mohammad Moslem Imani, Masoud Sadeghi, Habibolah Khazaie, Arezoo Sanjabi, Serge Brand, Annette Brühl, Dena Sadeghi Bahmani

**Affiliations:** ^1^Kermanshah University of Medical Sciences, Department of Orthodontics, Kermanshah, Iran; ^2^Kermanshah University of Medical Sciences, Medical Biology Research Center, Kermanshah, Iran; ^3^Kermanshah University of Medical Sciences, Sleep Disorders Research Center, Kermanshah, Iran; ^4^Kermanshah University of Medical Sciences, Students Research Committee, Kermanshah, Iran; ^5^University of Basel, Psychiatric Clinics, Center for Affective, Stress and Sleep Disorders, Basel, Switzerland; ^6^University of Basel, Department of Sport, Exercise and Health, Division of Sport Science and Psychosocial Health, Basel, Switzerland; ^7^Kermanshah University of Medical Sciences, Substance Abuse Prevention Research Center, Kermanshah, Iran; ^8^Tehran University of Medical Sciences, School of Medicine, Tehran, Iran; ^9^University of Basel, Psychiatric Clinics, Old Age Department, Basel, Switzerland

**Keywords:** cortisol, serum, plasma, sleep, obstructive sleep apnea syndrome, saliva, meta-analysis, pediatric and adult individuals

## Abstract

**Background:**

Obstructive sleep apnea syndrome (OSAS) may be associated with an increase in hypothalamic-pituitary-adrenocortical axis activity (HPA AA). We reviewed research comparing morning salivary and blood (serum and plasma) cortisol concentrations of individuals with OSAS to those of healthy controls.

**Methods:**

We made a systematic search without any restrictions of the PubMed/Medline, Scopus, Cochrane Library, and Web of Science databases for relevant articles published up to August 25, 2019.

**Results:**

Sixteen studies were analyzed in this meta-analysis; five studies compared morning salivary concentrations, five compared serum concentrations, four compared plasma cortisol concentrations, and two compared both salivary and plasma concentrations. In pediatric samples, compared to healthy controls, those with OSAS had significantly lower saliva morning cortisol concentrations (MD = -0.13 µg/dl; 95% CI: 0.21, -0.04; *P* = 0.003). In contrast, no significant differences were observed for serum cortisol concentrations, plasma cortisol concentrations, or salivary morning cortisol concentrations between adults with and without OSAS (*p* = 0.61, *p* = 0.17, *p* = 0.17).

**Conclusion:**

Cortisol concentrations did not differ between adults with OSAS and healthy controls. In contrast, morning salivary cortisol concentrations were lower in children with OSAS, compared to healthy controls. Given that a reduced HPA AA is observed among individuals with chronic stress, it is conceivable that children with OSAS are experiencing chronic psychophysiological stress.

## Introduction

Obstructive sleep apnea syndrome (OSAS) is one of the most common breathing disorders of sleep, with significant consequences for quality of life (1). Diagnosis of OSAS is made on the basis of nocturnal and diurnal clinical symptoms such as frequent collapses of the upper airway during sleep, resulting in intermittent nocturnal hypoxia and fragmented sleep (2–4). OSAS is defined by the Apnea–Hypopnea Index (AHI) as > 5 events per hour as measured by polysomnography for adults (5) and AHI > 1 events per hour for children (6). As regards prevalence rates, OSAS affects 2%–4% of those in middle age (2, 3); OSAS prevalence peaks between 40 and 65 years and decreases thereafter (7). In children and adolescents, prevalence rates range between 1.2% and 5.7% (8). The occurrence of OSAS is critical because both in children and adults it is associated with an increased risk of systemic comorbidities such as cardiovascular diseases, hypertension, metabolic syndrome, and cognitive impairments (2, 3). The most important epidemiological risk factors for OSAS are obesity and male gender (9, 10). OSAS plays an important role in the emergence and maintenance of hypertension, while snoring is a risk factor for hypertension independent of body mass index (BMI), AHI, or older age (11).

In pediatric samples, OSAS is associated with behavior problems, poor attention, cognitive deficits, poor school performance, long-term cardiovascular consequences (12), group A streptococcal infections (13), several related syndromes (14), snoring (15), impaired neurocognitive and behavioral development (16), and metabolic disturbances (17).

The physiological system known as the hypothalamic-pituitary-adrenocortical axis (HPA A) plays an important role in coping with acute and chronic psychophysiological demands (18). The key outcome of HPA A is cortisol, the major human glucocorticoid, assessed either in blood, urine, hair, or saliva (19). Briefly, HPA A activity (HPA AA) enables the organism to adapt quickly to stressors. Thus, a rapid rise and subsequent decrease in cortisol concentrations is considered a proxy for HPA AA capacity to adapt to a given context. In contrast, when stressors remain continuous, this capacity to adapt diminishes and cortisol secretion may remain either consistently high or consistently low. To illustrate, higher cortisol concentrations have been observed in individuals with major depressive disorders (18, 20–22), and in children with internalizing problems (23–25). In contrast, lower cortisol concentrations have been observed in individuals with posttraumatic stress disorders (PTSD) (26–29) and in women with postpartum depression disorder (30). From methodological and physiological points of view, saliva morning cortisol is an easy and non-invasive means of assessing HPA AA (cortisol awakening response (CAR) (31). Furthermore, HPA AA can be challenged either pharmacologically with the dexamethasone/corticotropin-releasing hormone (DEX/CRH-test (18, 20, 21): or non-pharmacologically with the Trier Social Stress Test (19, 32, 33).

The current work will provide an up-to-date synthesis of the differences in cortisol response/levels across those with and without OSAS.

To summarize, as a proxy for HPA AA, both higher and lower cortisol concentrations are associated with aspects of psychopathology when compared to healthy controls.

As regards HPA AA among individuals with OSAS, the pattern of findings is unclear. Among adults with OSAS, higher cortisol concentrations are associated with visceral obesity and the development of metabolic syndrome (34). An explanation for this observation is that intermittent hypoxia and sleep fragmentation are associated with sympathetic activation and catecholamine secretion. In parallel, concomitant repeated arousals also activate the HPA A (35). Thus, following Balbo et al. (36), it is likely that OSAS is associated with a lowered HPA AA; such a decrease appears to be mediated by immediate autonomic activation of the organism as a result of sleep loss, fractured sleep, and hypoxemia. It further follows that there is a reciprocal influence of sleep, and cortisol concentrations (37): while excessive HPA AA induces sleep fragmentation (38–40), sleep fragmentation in its turn raises cortisol levels (41). However, despite this apparent bidirectional association between cortisol concentrations and poor sleep among individuals with OSAS, research findings are mixed and inconsistent, at least for children and adolescents (42–44). Thus, when compared to healthy controls, children with OSAS show elevated HPA AA in both the morning and the evening (45). In contrast, low cortisol concentrations are associated with adenotonsillar hypertrophy and OSAS in children (46). The latter observation implies a lowered HPA AA in the longer term. In addition, there have also been inconsistent findings concerning the links between risk of OSAS and salivary (45, 47–49), serum (46, 50), and plasma (51–53) cortisol concentrations. Furthermore, given that cortisol plays an important role in lipid metabolism (54, 55), obesity might be a significant confounding variable in the inconsistent pattern of results (56). Given this, the aim of the present meta-analysis was to compare the morning salivary and blood (serum and plasma) cortisol levels of individuals with OSAS with those of healthy controls. We hold that for the following reasons the present meta-analysis is important: First, no meta-analyses on these topics have been performed so far in both children and adults with OSAS. Second, the pattern of results might have clinical and practical implications as regards further treatment interventions for children and adults with OSAS; more specifically, an altered HPA AA is associated with psychological and psychiatric issues; as such, identifying children and adults with OSAS and an altered HPA AA might help to prevent further psychological and psychiatric issues. Third, reporting results from both children and adults at a glance enables the reader to get a quick and accurate overview, if and to what extent OSAS and HPA AA might differ between children and adults. Fourth, the present pattern of results may instigate further research on these two topics.

## Materials and Methods

The review process followed the Preferred Reporting Items for Systematic Reviews and Meta-Analyses (PRISMA) statement guidelines (57).

### Search Strategy

One of the authors (MS) systematically searched four databases, namely PubMed/Medline, Scopus, Cochrane Library, and Web of Science, for articles published in English up to August 25, 2019 with no restrictions. The searched keywords were (“sleep apnea” or “obstructive sleep apnea” or “OSA” or “obstructive sleep apnea syndrome” or “OSAS”) and (“cortisol”). We also manually searched the articles’ reference lists (original and review articles) for publications related to our topic.

### Eligibility Criteria

*Inclusion criteria:* I) studies with case-control design without age, sex, or BMI restrictions, II) OSAS was defined as AHI > 5 events/h in adults, and AHI > 1 events/h in children, III) OSAS was diagnosed on the basis of polysomnography, IV) controls had mild OSAS and no other systematic diseases, V) OSAS patients (moderate to severe OSAS) had no other systematic diseases, VI) studies reporting pretreatment morning salivary, serum, and/or plasma levels of cortisol (around 7–10 AM), and (II) studies having sufficient data to calculate the mean difference (MD) and 95% confidence interval (CI).

*Exclusion Criteria*: I) Studies including OSAS patients with a history of chronic airway disease, cerebrovascular disease, chronic cardiac failure, malignancies, endocrine disease, and mental disorder (depression and anxiety), II) studies with irrelevant or insufficient data to calculate the MD and 95% CI, III) review articles, letters to editors, conference papers, and book chapters, IV) studies without a control group, V) studies reporting urinary or hair levels of cortisol, VI) studies reporting controls with AHI > 5 events/h in adults, and AHI > 1 events/h in children, and VII) studies reporting data overlapping with other studies.

### Study Selection

Two of the authors, MMI and MS, read the titles and abstracts of the retrieved studies. They then selected the relevant studies and another of the authors, AS, retrieved the full-texts of the articles and excluded several full-texts based on the exclusion criteria set out above. If two studies had overlapping data (same data carried out from the same samples), we selected the study with the more recent publication year.

### Data Extraction

MMI and SB independently extracted the data from each study included in the meta-analysis. If there was a disagreement between the two authors this was resolved by the third author (AS). The data extracted for the present meta-analysis included the first author, publication year, country of study, ethnicity of individuals in each study, percentage of males, mean age, BMI and AHI of both groups (OSAS patients and controls), and the method of measurement of cortisol level.

### Quality Assessment

One author (MS) evaluated the quality of the studies included in the meta-analysis using the Newcastle-Ottawa Scale (NOS) (58). Total score for every study was nine.

### Statistical Analyses

The data analysis was performed by one of the authors (MS). Review Manager 5.3 software (RevMan 5.3) was used to calculate the crude MD and 95% CI; the significance of the pooled MD was assessed by the Z test. Heterogeneity across the studies was evaluated using both the Cochrane Q test (59) and I^2^ metric with a range of 0 to 100% (60). In addition, when P-value <0.1 and I^2^>50% showed a statistically significant heterogeneity the analysis was performed by the random-effect model to evaluate the pooled ORs and CI values. Otherwise, we used the fixed-effect model.

For this meta-analysis, subgroup analysis was performed based on ethnicity, method, and mean BMI to explore potential heterogeneity. Another method of evaluating heterogeneity across studies is meta-regression analysis. This analysis indicates whether there are significant associations of study periods, number of individuals, male percentage ratio, mean BMI, and mean age with the pooled MD.

The results of Begg’s and Egger’s tests were analyzed using the Comprehensive Meta-Analysis version 2.0 software (CMA 2.0). The Begg’s funnel plot illustrates the standard error (SE) of the log (MD), and the precision of each study is plotted against its log (MD) (61). In addition, Egger’s test gives the linear regression between the precision of the studies and the standardized effect (62). To estimate the consistency/stability of the results, sensitivity analyses, namely the “cumulative analysis” and “one study removed”, were used. A p-value (two-tailed) smaller than 0.05 was taken to indicate a statistically significant difference.

In some studies included in this meta-analysis the cortisol values were reported by SE and we changed these to standard deviation (SD), $\left(SE=\frac{SD}{\sqrt{N}};N=number\;of\;individuals\right)$. The cortisol levels in the saliva, serum, and plasma were reported in microgram per deciliters (µg/dl). Individuals with BMI >30 kg/m^2^ were considered obese (47).

## Results

**Figure 1:**
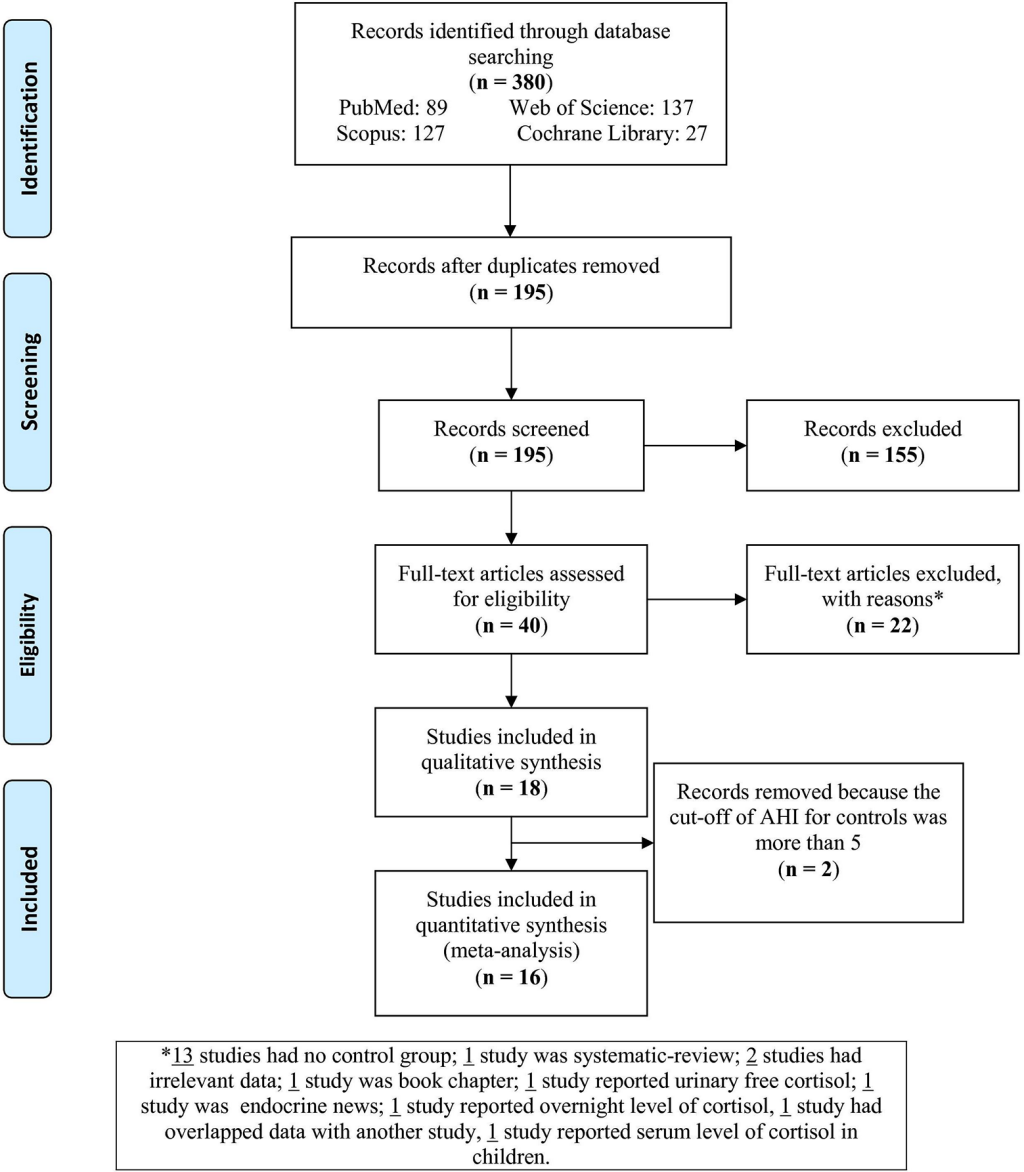
Flow chart of the study selection.

The flow chart (**Figure 1**) sets out the selection process. The search of databases yielded 380 articles; of these, 185 were duplicates and therefore removed. Of the remaining 195 records, 155 were excluded as irrelevant to the present topic. Next, full texts of the 40 remaining articles were assessed for eligibility. Of these, 22 articles were excluded for the following reasons: 15 had no control group, one was a systematic review, one was a book chapter, one reported urinary free cortisol, one focused on other endocrine outcomes, one reported overnight level of cortisol, one had data overlapping with another study, and one study reported serum level of cortisol in children. Of the remaining 18 studies, one study of plasma cortisol levels used individuals with AHI < 9 (37) and one with AHI < 15 (63) as controls; these two studies were therefore also excluded. This left 16 studies to be analyzed in this meta-analysis.

**Table 1** presents the characteristics of the 16 studies included in the present meta-analysis (45, 47–53, 56, 64–70). The studies had been published between 1996 and 2019. Five studies reported salivary cortisol concentrations (45, 47–49, 70), five studies reported serum cortisol concentrations (50, 65, 67–69), four reported plasma cortisol concentrations (53, 56, 64, 66), and two reported both salivary and plasma cortisol concentrations (51, 52). Ten studies were performed with Caucasians (45, 47, 50, 52, 56, 64–67, 69), four studies sampled Asians (48, 49, 53, 68), and two studies sampled mixed ethnicities (51, 70). Fourteen out of 16 studies assessed adult samples, the other two assessed pediatric samples (45, 48). **Table 1** reports other study characteristics including sample size, BMI, age, AHI, and the method of cortisol analysis [immunoenzymatic analysis; radioimmunoassay; electrochemiluminescence immunoassay analyzer (ECLIA)].

**Table 1:** Characteristics of the studies included in this meta-analysis.

Author, Publication year	Country	Ethnicity	Number of (OSAS patients/controls)	Male percentage (%) (OSAS patients/controls)	MeanBMI,kg/m2(OSAS patients/controls)	Mean age, year (OSAS patients/controls)	Mean AHI, events/h (OSAS patients/controls)	Method of assessment	Sample
Entzian et al. 1996 (64)	Germany	Caucasian	10/10	90/50	Obese/NA	53.5/Range: 19 to 29	Range: ≥5/<5	Immunoenzymatic	Plasma
Lanfranco et al. 2004 (65)	Italy	Caucasian	15/30	100/100	39.2/31.2	43.5/39.7	53.4/2.5	Radioimmunoassay	Serum
Feng et al. 2006 (53)	China	Asian	33/11	84.8/81.8	27.6/26.6	51.6/40.6	44.5/3.5	Radioimmunoassay	Plasma
Dadoun et al. 2007 (52)	France	Caucasian	15 & 9/77 & 35	100/100	37.8/28.8	44.5/42.4	Range: ≥5/<5	Radioimmunoassay	Saliva & Plasma
Vgontzas et al. 2007 (66)	Greece	Caucasian	16/28	100/100	38.4/31.1	46.6/42.8	48.7/0.95	Radioimmunoassay	Plasma
Barceló et al. 2008 (67)	Spain	Caucasian	22/23	100/100	31/25	50/48	48/3	ECLIA	Serum
Carneiro et al. 2008 (51)	Brazil	Mixed	16/13	100/100	46.9/42.8	40.1/38.8	65.7/3.2	NA	Saliva & Plasma
Panaree et al. 2011 (68)	Thailand	Asian	39/24	61.5/100	28.5/21.5	47.2/32.3	22.3/1.6	ECLIA	Serum
Park et al. 2013 (48)	Korea	Asian	27/53	66.7/40.3	18.1/18.6	7.3/6.9	Range: ≥5/<5	Immunoenzymatic	Saliva
Patacchioli et al. 2014 (45)	Italy	Caucasian	14/20	50/40	15.8/15.9	4.5/5.3	Range: ≥5/<5	Immunoenzymatic	Saliva
Yildirim et al. 2015 (69)	Turkey	Caucasian	25/25	48/52	46.9/39.9	46.7/43.8	24.3/2.3	NA	Serum
Ghiciuc et al. 2016 (47)	Romania	Caucasian	10/7	100/100	32.3/32.2	53/51	63.5/2.6	Immunoenzymatic	Saliva
Kritikou et al. 2016 (56)	Greece	Caucasian	35/37	54.3/40.1	28.5/27.2	55.5/53.3	38.5/2.3	Radioimmunoassay	Plasma
Madaeva et al. 2018 (50)	Russia	Caucasian	37/14	100/100	34.2/28.2	Range: 46–55 (Matched/Matched)	Range: ≥5/<5	Radioimmunoassay	Serum
Farabi et al. 2019 (70)	USA	Mixed	12/6	0/0	Range: ≥30 to ≤40	20–39	Range: ≥5/<5	Immunoenzymatic	Saliva
Yan et al. 2019 (49)	China	Asian	46/12	78.7/66.7	28.3/24.8	45.4/46.2	42.9/2.9	Immunoenzymatic	Saliva

ECLIA, Electrochemiluminescence immunoassay analyzer; NA, Not available; OSAS, Obstructive sleep apnea syndrome; AHI, Apnea-hypopnea index.

### Pooled Analyses

#### Moring Saliva Cortisol Concentrations

**Figure 2:**
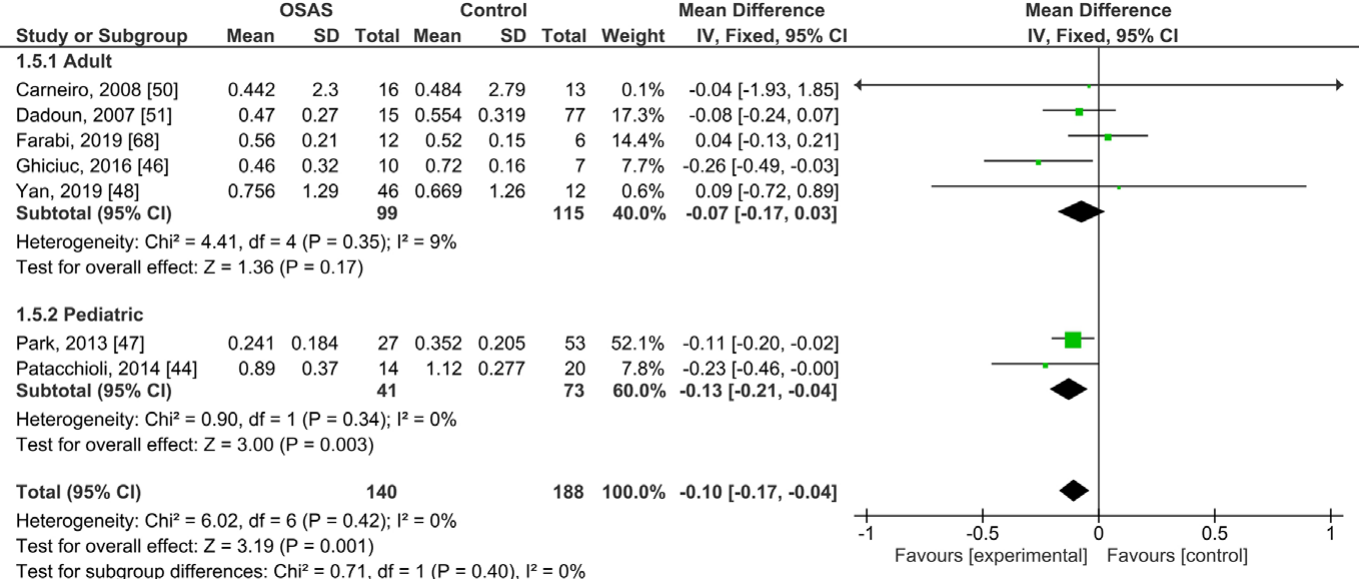
Forest plot of random-effects analysis of salivary cortisol levels in adult and pediatric obstructive sleep apnea syndrome (OSAS) versus the control group. The diamond shape indicates the pooled mean difference (MD). Each black box represents a point estimate of each study and also gives a representation of the study size (the bigger the box, the more participants in the study). A horizontal line representing the 95% confidence intervals (CIs) of the study result, with each end of the line representing the boundaries of CI. SD, Standard deviation.

Five studies compared morning salivary cortisol concentrations of OSAS adults and those of healthy controls; there were no significant mean differences {pooled MD was -0.07 µg/dl [95% CI: -0.17, 0.03; *P* = 0.17; I^2^ = 9% (P_h_ or P_heterogeneity_ = 0.35)]}. Two studies compared the morning salivary cortisol concentrations of pediatric samples with OSAS and healthy controls and found significantly lower salivary cortisol concentrations in those with OSAS {-0.13 µg/dl [95% CI: -0.21, -0.04; P = 0.003; I^2^ = 0% (P_h_ = 0.34)]; see **Figure 2**}.

#### Serum Cortisol Concentrations

**Figure 3:**
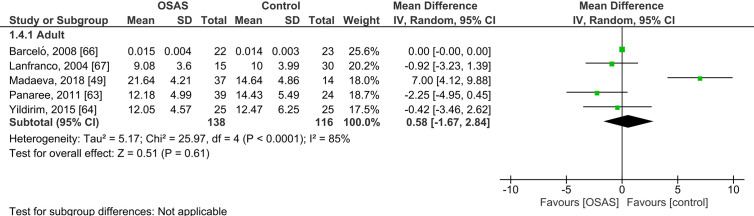
Forest plot of random-effects analysis of serum cortisol levels in adult obstructive sleep apnea syndrome (OSAS) versus the control group. The diamond shape indicates the pooled mean difference (MD). Each black box represents a point estimate of each study and also gives a representation of the study size (the bigger the box, the more participants in the study). A horizontal line representing the 95% confidence intervals (CIs) of the study result, with each end of the line representing the boundaries of CI. SD, Standard deviation.

Five studies compared serum cortisol concentrations of adults with OSAS and healthy controls (see **Figure 3**). The pooled MD was 0.58 µg/dl [95% CI: -1.67, 2.84; *P* = 0.61; I^2^ = 85% (P_h_ < 0.0001)] for adults with OSAS. Thus, serum cortisol concentrations did not significantly differ between those with and those without OSAS.

#### Plasma Cortisol Concentrations

**Figure 4:**
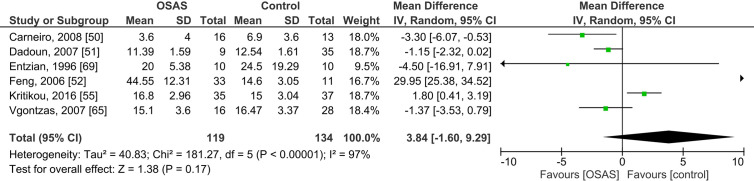
Forest plot of random-effects analysis of plasma cortisol levels in adult obstructive sleep apnea syndrome (OSAS) versus the control group. The diamond shape indicates the pooled mean difference (MD). Each black box represents a point estimate of each study and also gives a representation of the study size (the bigger the box, the more participants in the study). A horizontal line representing the 95% confidence intervals (CIs) of the study result, with each end of the line representing the boundaries of CI. SD, Standard deviation.

Six studies compared plasma cortisol concentrations of adults with OSAS and healthy controls and found no significant mean differences (see **Figure 4**): the pooled MD was 3.84 µg/dl [-1.60, 9.29; *P* = 0.17; I^2^ = 97% (P_h_ < 0.00001)].

#### Subgroup Analysis

Next, for the studies of adults with OSAS, a series of subgroup analyses was performed with ethnicity, method of cortisol analysis, and mean BMI as independent variables and salivary, serum, and plasma cortisol concentrations as dependent variables (**Table 2**). Among Caucasian samples, adults with OSAS had significantly lower morning salivary cortisol concentrations than healthy controls. In contrast, among Asian samples, plasma cortisol concentrations were significantly higher in adults with OSAS than in healthy controls. Last, again compared to healthy controls, significantly lower plasma cortisol concentrations were observed among mixed ethnicities with OSAS, and among participants with a mean BMI > 30 kg/m^2^.

**Table 2:** Subgroup analysis based on ethnicity and method on salivary, serum, and plasma levels of cortisol in adult obstructive sleep apnea syndrome.

Subgroup analysis of salivary level (n)	OR (95%CI), I^2^ (%), P_h_	Subgroup analysis of serum level (n)	OR (95%CI), I^2^ (%), P_h_	Subgroup analysis of plasma level (n)	OR (95%CI), I^2^ (%), P_h_
Overall (5)	-0.07 (-0.17, 0.03), 9, 0.35	Overall (5)	0.58 (-1.67, 2.84), 85, <0.0001	Overall (6)	3.84 (-1.60, 9.29), 97, <0.00001
Ethnicity		Ethnicity		Ethnicity	
Caucasian (2)	-0.14 (-0.27, -0.015), 35, 0.21	Caucasian (4)	1.26 (-1.46, 3.99), 87, <0.0001	Caucasian (4)	-0.29 (-2.32, 1.74), 75, 0.007
Asian (1)	0.09 (-0.72, 0.89)	Asian (1)	-2.25 (-4.95, 2.62)	Asian (1)	29.99 (25.38, 34.52)
Mixed (2)	0.04 (-0.13, 0.21), 0, 0.93	Mixed (0)	NA	Mixed (1)	-3.30 (-6.07, -0.53)
Method		Method		Method	
Immunoenzymatic (3)	-0.08 (-0.32, 0.16), 54, 0.11	Immunoenzymatic (0)	NA	Immunoenzymatic (1)	-4.5 (-16.91, 7.91)
Radioimmunoassay (1)	-0.08 (-0.24, 0.07)	Radioimmunoassay (2)	2.99 (-4.77, 10.75), 94, <0.0001	Radioimmunoassay (4)	6.78 (-0.03, 13.59), 98, <0.00001
ECLIA (0)	NA	ECLIA (2)	-0.70 (-2.75, 1.34), 63, 0.10	ECLIA (0)	NA
Mean BMI, kg/m^2^		Mean BMI, kg/m^2^		Mean BMI, kg/m^2^	
>30 (4)*	-0.07 (-0.17, 0.03), 30, 0.24	>30 (3)	1.86 (-3.09, 6.81), 90, <0.0001	>30 (3)	-1.45 (-2.42, -0.49), 0, 0.37
≤30 (1)**	0.09 (-0.72, 0.89)	≤30 (2)	-0.70 (-2.75, 1.34), 63, 0.10	≤30 (2)	15.79 (-11.80, 43.37), 99, <0.00001

BMI, Body mass index; OR, Odds ratio; ELICA, Electrochemiluminescence immunoassay analyzer; NA, Not available; P_h_, P_heterogeneity._ The data didn't show statistically significant values (P-value < 0.05). *The mean BMI of the two groups (patients and controls) was >30 kg/m^2^. **The mean BMI of the one group (patients or controls) or both groups was ≤30 kg/m^2^.

#### Sensitivity Analysis

The “cumulative analysis” and “one study removed” did not affect the pattern of results in these studies; the consistency and stability of the pattern of results were therefore confirmed. For plasma cortisol concentrations, we excluded one study with outlier data (53) but the pattern of results remained unchanged [MD = -0.89; 95% CI: -2.83, 1.06; *P* = 0.37; I^2^ = 76% (P_h_ = 0.002)]. For serum cortisol concentrations, we excluded two studies with outlier data (50, 67); while the pattern of results remained unchanged [MD = -1.22; 95% CI: -2.74, 0.30; *P* = 0.12; I^2^ = 0% (P_h_ = 0.64)], heterogeneity decreased to 0%.

#### Quality Assessment

The quality score of each study included in the meta-analysis is given in **Table 3**. Among 16 studies, 13 studies had a high quality (score ≥ 7).

**Table 3:** Quality assessment scores of the studies involved in the meta-analysis.

The first author (year)	Selection	Comparability	Exposure	Total points
Entzian, 1996 (64)	**	–	***	5
Lanfranco, 2004 (65)	***	**	***	8
Feng, 2006 (53)	***	**	***	8
Dadoun, 2007 (52)	***	**	***	8
Vgontzas, 2007 (66)	***	**	***	8
Barceló, 2008 (67)	***	**	***	8
Carneiro, 2008 (51)	**	**	***	7
Panaree, 2011 (68)	**	*	***	6
Park, 2013 (48)	**	**	***	7
Patacchioli, 2014 (45)	***	**	***	8
Yildirim, 2015 (69)	**	**	***	7
Ghiciuc, 2016 (47)	***	**	***	8
Kritikou, 2016 (56)	***	**	***	8
Madaeva, 2018 (50)	***	**	***	8
Farabi, 2019 (70)	**	*	***	6
Yan, 2019 (49)	**	**	***	7

Each asterisk denotes 1 point.

#### Publication Bias

**Figure 5:**
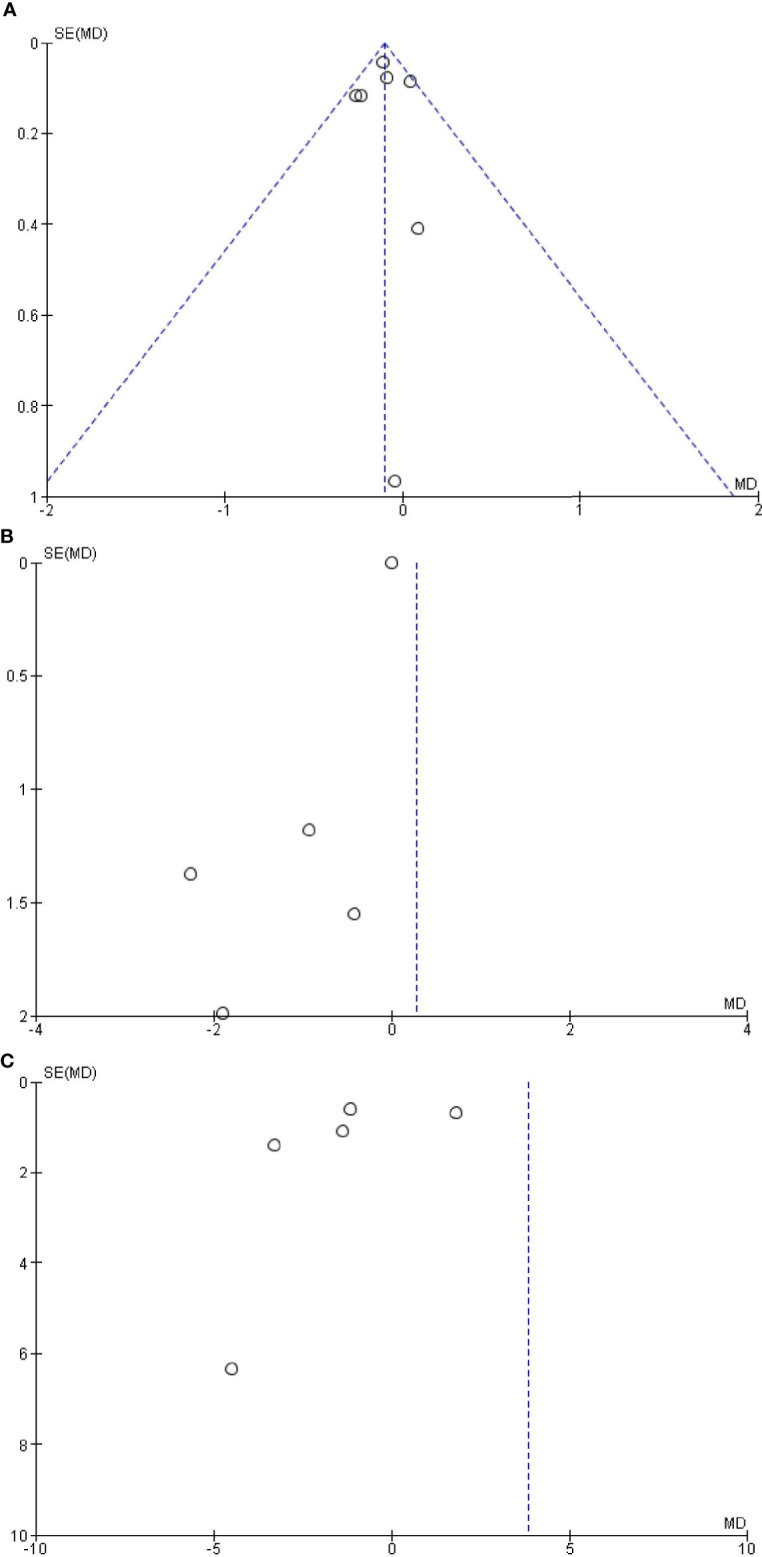
Funnel plot of the **(A)** salivary, **(B)** serum, and **(C)** plasma levels of cortisol in adult obstructive sleep apnea syndrome versus the control group. Black circles represent imputed studies. Open circles represent observed studies. SE, Standard error; MD, Mean difference.

The Begg’s and Egger’s tests did not identify any publication bias across the studies of adults with OSAS [saliva: Egger’s test (*P* = 0.991) and Begg’s test (*P* = 0.851); serum: Egger’s test (*P* = 0.832) and Begg’s test (*P* = 0.851); plasma: Egger’s test (*P* = 0.451) and Begg’s test (*P* = 0.347); **Figure 5**].

#### Meta-Regression

**Table 4** reports the associations of year of publication, the number of individuals, the male percentage, the mean BMI, and mean age with the pooled mean differences of the saliva, serum, and plasma cortisol concentrations. There was a significant positive correlation between mean age (OSAS patients/control) and the pooled mean difference of the plasma cortisol concentrations (r = .966; *P* = 0.008). For all other variables, correlation coefficients were trivial.

**Table 4:** Meta-regression analysis, based on several variables [year of publication, number of individuals, male percentage, mean body mass index (BMI), mean age], for salivary, serum, and plasma levels of cortisol in adult obstructive sleep apnea syndrome patients compared to controls.

Year of publication	R	Adjusted R^2^	*P*	Male percentage (OSAS/control)	R	Adjusted R^2^	*P*	Mean age (OSAS/control)	R	Adjusted R^2^	*P*
Saliva	0.382	-0.025	0.398	Saliva	0.373	-0.033	0.41	Saliva	0.227	-0.186	0.666
Serum	0.576	0.164	0.232	Serum	0.238	-0.179	0.65	Serum	0.172	-0.213	0.745
Plasma	0.134	-0.228	0.801	Plasma	0.052	-0.33	0.934	Plasma	0.966	0.911	0.008
**Number of individuals**	**R**	**Adjusted R^2^**	***P***	**Mean BMI (OSAS/control)**	**R**	**Adjusted R^2^**	***P***				
Saliva	0.205	-0.149	0.659	Saliva	0.511	0.076	0.301				
Serum	0.352	-0.095	0.494	Serum	0.47	-0.038	0.424				
Plasma	0.213	-0.193	0.685	Plasma	0.51	0.013	0.38				

The correlation coefficient (R) of a model (variables x and y) takes values between −1 and 1. It describes how x and y are correlated. The adjusted R2 shows how well terms fit a curve or line, but adjusts for the number of terms in a model. Also, the adjusted R2 increases only if the new term improves the model more than would be expected by chance. It decreases when a predictor improves the model less than would be expected by chance.

## Discussion

The key findings of the present meta-analysis were as follows. For pediatric samples with OSAS, morning salivary cortisol concentrations were significantly lower than those of healthy controls. However, no significant mean differences in salivary, serum or plasma cortisol concentrations were observed between adults with and those without OSAS. Next, the sensitivity analysis confirmed the previous results for the serum and plasma cortisol levels, as the results of the sensitivity analysis had lower heterogeneity than previous results. Subgroup analyses showed that, compared to healthy controls, adult Caucasians with OSAS had significantly lower salivary cortisol concentrations while adult Asians with OSAS had significantly higher salivary cortisol concentrations. In addition, compared to healthy controls, lower plasma cortisol concentrations were observed both in adult samples with mixed ethnicities and in samples with participants with > 30 kg/m^2^. Last, in adults with OSAS, greater age was associated with higher plasma cortisol concentrations.

The present findings add to the current literature in an important way: the pattern of results indicates a complex association between HPA AA and the occurrence of OSAS in pediatric and adult samples. The findings are clinically important: they imply that children with OSAS may be under continuous psychophysiological stress. It follows that the results are also of practical importance because pediatricians treating children with poor daytime performance (low mood; poor school performance; higher sedentary behavior) should also assess those children for sleep- and breathing-related issues. Relatedly, lower cortisol values may be indicative of a longer-term, chronic psychophysiological burden.

Plasma, serum and morning saliva cortisol concentrations in adults with OSAS did not significantly differ between individuals with and those without OSAS. This pattern of results is in line with the findings reported in a previous systematic review of studies of adults (71). Also Tomfohr et al. (71) were unable to identify statistically significant mean differences of cortisol concentrations between adults with and without OSAS. Furthermore, these authors mentioned that possible confounders such as sampling, sample sizes, age, and BMI, along with the inconsistent control of such confounders might have biased the overall pattern of results (71). For the following reasons this pattern we identified is surprising and merits particular attention. OSAS is associated with impaired sleep (5, 72–76); more specifically, intermittent hypoxia and sleep fragmentation appear to be associated with sympathetic activation and catecholamine secretion, while concomitant repeated arousals also activate the HPA A (35). In addition, impaired sleep is associated with an up-regulation of HPA AA (39, 40). Following Balbo et al. (36), it is also likely that OSAS is associated with a reduction in HPA AA, given that sleep loss, fractured sleep and hypoxemia appear to lead to an immediate autonomic activation of the organism. As mentioned, it further follows that the direction of influence between sleep and cortisol concentrations is bi-directional (37); while excessive HPA AA induces sleep fragmentation (38–40), sleep fragmentation in turn increases cortisol levels (41). Relatedly, the hyperarousal model of insomnia (77) claims that psychological issues lead to an up-regulation of physiological factors such as cortisol and orexin to increase wakefulness, most probably to cope with these issues. However, the result of such an up-regulation is a disrupted and fragmented sleep, which in terms of a negative feedback impacts again on wakefulness. Given this, one would have expected altered cortisol concentrations in individuals with OSAS. However, the pattern of results was more complex and was not fully consistent with this expectation. OSAS and cortisol concentrations were virtually unrelated. In the absence of direct evidence of the underlying psychophysiological mechanisms we offer the following admittedly speculative interpretation.

First, children and adults with OSAS differ in their respective salivary morning cortisol concentrations. Therefore, developmental stage should be considered when discussing HPA AA in individuals with OSAS.

Second, differences in HPA AA have been observed for salivary morning cortisol concentrations among pediatric samples. It follows that the type of cortisol sampling, including the laboratory processes to analyze cortisol concentrations (immunoenzymatic analysis; radioimmunoassay; ECLIA) may obscure the overall pattern of associations among adults.

Third, results from the meta-regression showed that neither gender nor BMI were associated with higher plasma cortisol concentrations, but greater age was (though not with salivary morning or serum cortisol concentrations; **Table 4**). It follows that age may be a confounder. It is worth noting that the present findings are at odds with the results reported by Tenk et al. (78). At least among individuals with obesity, HPA AA did not increase with BMI; instead it decreased with age. This might be critical as a lower cortisol concentration has been found to be associated with lower lipid metabolism (54, 55) and thus with increasing BMI.

Fourth, the subgroup analysis revealed that ethnicity might bias the results. Compared to healthy controls, Caucasian adults with OSAS had lower salivary morning cortisol concentrations, Asian adults with OSAS had higher plasma cortisol concentrations (53), while lower plasma cortisol concentrations were found in a study sampling mixed ethnicities (51). A thorough literature search did not identify any studies comparing cortisol concentrations, OSAS and ethnicity. It follows that the present findings are novel, while there is currently no explanation for these results. It also follows that future studies might take ethnicity into consideration as a possible confounder.

Fifth, higher BMI was associated with lower cortisol concentrations (see **Table 2**). This accords with Vgontzas et al.’s (66) study; obesity was related to cortisol levels lower than those of normal-weight controls. In contrast, the present results and those of Vgontzas et al. (66) are not reflected in two recent meta-analyses (54, 78) both of which concluded that BMI is either not associated with cortisol concentrations, or that cortisol concentrations decrease with age. In contrast, significant increases in BMI and AHI have been reported in adults with OSAS (51, 79, 80).

Sixth, Stalder et al. (31) noted, in their paper on expert consensus guidelines, that salivary cortisol samples should be taken with strict reference to time of awakening. It is therefore possible that studies of salivary morning cortisol concentrations lacked precision in their sampling protocols and thus produced biased estimates of cortisol concentrations.

To summarize, the pattern of results identified in the present meta-analysis indicate a complex but unexplained association between HPA AA and the occurrence of OSAS among adults, and to some extent also in pediatric samples with the same disorder. This latter group also had lower salivary morning cortisol concentrations than healthy controls.

A lowered cortisol secretion is associated with psychophysiological issues. For example, adults with PTSD have a lower HPA AA (26–28). Similarly, women with post-partum depression 12 weeks after delivery were already displaying significantly lower cortisol concentrations at least 12 weeks before delivery (30). By way of explanation, it is possible that chronic stress produces a complete reduction and depletion of cortisol production; at a behavioral level, such individuals may show clear signs of depression, lack of energy, vigor and motivation, along with a lack of initiative and self-assertiveness. The same process may occur in children with OSAS and thereby suffering from chronic stress which is reflected in dramatically reduced HPA AA (81).

The question might arise as to whether a reduced HPA AA is either a dysfunctional or a functional neurophysiological adaption to stress. As regards women with postpartum depression, at least from an evolutionary point of view post-partum depression could be understood as a means of avoiding further loss of energy and of seeking a greater investment and engagement from their social environment (82, 83). For PTSD, a reduced HPA AA might protect against further impulsivity and harmful behavior, typically observed in individuals with PTSD. Likewise, for children with OSAS, one might hypothesize that a reduced HPA AA may protect against additional loss of energy, thus providing a degree of protection.

Next, severe OSAS is associated with low morning serum cortisol concentrations in children with OSAS and tonsillar hypertrophy (46). In contrast, no such association has been observed among children with OSAS who have typically developing tonsils (46). Therefore, one might speculate that children with OSAS and with tonsillar hypertrophy are suffering from chronic (physiological) stress, as reflected in reduced HPA AA.

An additional set of associations may be operating for the following reasons. First, pediatric OSAS is a serious health concern, thus, latent and unassessed physiological issues might interfere with both the HPA AA and OSAS (84). Second, children with tonsillar hypertrophy are at greater risk of OSAS, and this risk is particularly high among obese children (85). Third, OSAS appears to be associated with increased psychophysiological stress. Fourth, despite improvements in objective sleep parameters following adenotonsillectomy, the risk of residual symptoms of OSAS is higher in obese children (86). Fifth, lower cortisol concentrations are associated with lowered lipid metabolism (54, 55) and thus with an increasing BMI.

In brief, it is conceivable that in pediatric populations higher OSAS, lower cortisol concentrations, lower lipid metabolism and higher BMI are intertwined in a vicious circle.

While we were unable to identify any publication bias, there are nonetheless limitations that argue against generalization of the present conclusions. First, the number of studies was small especially with respect to samples of children; a greater number of studies and larger samples might have yielded another pattern of results. Second, sample sizes were often also small. Third, methodological issues such as sampling method, sampling time, and different methods of cortisol analysis might have biased the entire pattern of results. Fourth, sex, age and BMI could have affected the pattern of results both between and within individual studies. Fifth, it was not possible to calculate adjusted mean differences. Sixth, the present results are based on studies that included only one measurement of cortisol. This may have obscured the overall pattern of results and rendered the conclusions more inaccurate. Given this, future studies might give more thorough consideration to the contextualization of stress. Seventh, the use of Continuous Positive Air Pressure (CPAP) devices is the gold standard in the treatment of OSAS; given this, it would also be of clinical relevance to systematically investigate whether or to what extent cortisol concentrations change following the use of CPAP. An inspection *via* PubMed of the current state of research on this topic yielded 27 publications (up to August 2020), though, no systematic review or meta-analysis has been carried out so far. For the following three reasons we took the decision not to include studies on the possible impact of CPAP on the HPA AA.

First, the practical and clinical importance of the use of CPAP and its possible impact on HPA AA is such as to deserve a thorough description and discussion in its own right. Second and relatedly, 27 studies should provide the basis for running a systematic review and meta-analysis of its own. Third, a thorough inspection of such studies should also include as a research question the extent to which the use of CPAPs alters any associations between HPA AA and sleep patterns.

## Conclusions

The associations between OSAS and the HPA AA are far from straightforward or conclusive. Against expectations, saliva, serum and plasma concentrations as proxies for HPA AA did not differ between adults with OSAS and healthy controls. However, pediatricians treating children with low daytime performance might suspect undetected and untreated OSAS. In addition, the assessment of salivary morning cortisol as an easy, non-invasive and low-cost measure of HPA AA might also aid in diagnosis of OSAS and in monitoring its treatment. The present meta-analysis provides highly synthetic evidence for objectively evaluated associations between child and adult OSAS and their HPA AA activity as measured by changes in morning saliva, plasma and blood cortisol levels. One of the significant merits of this review is that it considers the role of age, methods used for measuring cortisol levels, BMI, ethnicity, and presence of concomitant psychiatric disorders.

## Data Availability Statement

The original contributions presented in the study are included in the article/supplementary material; further inquiries can be directed to the corresponding author.

## Author Contributions

Conceptualization, MMI; Formal analysis, MS; Methodology, MMI and MS; Supervision, MMI and SB; Validation, MS, HK, AB and SB; Visualization, HK and AS; Writing-original draft, SB, MS, DSB, AB; Writing-review and editing, MS, SB, AB and DSB. All authors contributed to the article and approved the submitted version.

## Funding

This work was performed in partial fulfillment of the requirements for a doctorate degree in General Dentistry (AS), in Faculty of Dentistry, Kermanshah University of Medical Sciences, Kermanshah, Iran. This study was funded by the Research Council of Kermanshah University of Medical Sciences (Grant Number: 980687). In addition, we thank Nick Emler (University of Surrey, Surrey UK) for proofreading the manuscript.

## Conflict of Interest

The authors declare that the research was conducted in the absence of any commercial or financial relationships that could be construed as a potential conflict of interest.

## Abbreviations

AHI, Apnea–Hypopnea Index; BMI, Body mass index; CAR, Cortisol awakening response; CI, Confidence interval; CMA, Comprehensive Meta-Analysis; CPAP, Continuous Positive Air Pressure; DEX/CRH, Dexamethasone/corticotropin-releasing hormone; ECLIA, Electrochemiluminescence immunoassay analyzer; HPA A, Hypothalamic-pituitary-adrenocortical axis; HPA AA, Hypothalamic-pituitary-adrenocortical axis activity; MD, Mean difference; NOS, Newcastle-Ottawa Scale; OSAS, Obstructive sleep apnea syndrome; PRISMA, Preferred Reporting Items for Systematic Reviews and Meta-Analyses; PTSD, Posttraumatic stress disorder; RevMan, Review Manager; SD, Standard deviation; SE, Standard error.
